# Supplementary material for: Quantitative evidence for dimorphism suggests sexual selection in the maxillary caniniform process of Placerias hesternus
Source: PLoS One. 2024 May 31;19(5):e0297894. doi: 10.1371/journal.pone.0297894 (PMC11142433; doi:10.1371/journal.pone.0297894)
Supplement: Attachment — Submitted filename: PintoEtAl_Placerias_Review.docx [file pone.0297894.s005.docx]

**Review – Pinto et al., “Quantitative evidence for dimorphism suggests sexual selection in the maxillary caniniform process of *Placerias hesternus***

**Review by Corwin Sullivan**

Sexual dimorphism has been repeatedly suggested in various dicynodont synapsids, and Pinto et al. have produced a worthy addition to this literature by making a strong case for dimorphism in the caniniform process of *Placerias hesternus*. The manuscript is well argued and well illustrated, and the evidence adduced for interpreting the caniniform process as a sexually dimorphic structure is compelling. On this basis, I’m delighted to recommend publication of the paper in *PLOS ONE*, following some minor revisions.

The paper ends by suggesting that the caniniform process was not just a dimorphic structure, but more specifically a weapon as opposed to an ornamental feature. This is perfectly plausible, but I think the argument could be greatly strengthened by more explicitly considering *how* one *Placerias* could have used its caniniform process to attack another. How well would the caniniform process have lent itself, for example, to stabbing and/or slashing? Another point to be made in this connection is that, if the caniniform process took over the feeding and display functions of the tooth as suggested earlier in the Discussion, it may have taken over a combat-related function as well.

The authors suggest that the morph with the large caniniform process was likely male rather than female, but are somewhat noncommittal on this point. I think a more definite case could be made that the large-caniniform morph was almost certainly the male, based not only on analogy to modern mammals but also on the distribution of sexually dimorphic armament more or less throughout the animal kingdom. Furthermore, males almost always have less investment in the offspring than females, and therefore are much more likely to have a strong incentive to maximise their mating opportunities by engaging in violent competition.

The manuscript devotes too much space, attention and analysis to cases, like that of the distance between the anterior tip of the premaxilla and the nasal process, in which the distribution of data for a measurement appears somewhat bimodal when inspected visually but statistical support for bimodality is lacking. The visual impression of bimodality is never very clear, at least to me, and in my opinion deserves to be mentioned only briefly. This aspect of the paper gives the impression that the authors are trying to wring (even) more dimorphism out of their data than is really there.

It seems odd to me that the “dorsoventral length”, “mediolateral length” and “proximal anteroposterior length” measurements that lie at the core of the quantitative analysis of the maxilla’s proportions are actually not strictly dorsoventral, mediolateral and anteroposterior, but rather are all oblique measurements between specific landmarks (whereas I would expect a dorsoventral measure of an element to be the distance along the dorsoventral axis from the dorsalmost point to the ventralmost point, as if the measurement were being taken with giant calipers). An easy fix for this would be to simply rename the measurements, so that “dorsoventral length”, for instance, could become something like “jugal-caniniform length”.

The paper is nicely illustrated, and the writing is generally serviceable but sometimes vague and difficult to follow. There are a lot of sentences that contain grammatical slip-ups and/or don’t quite say what they’re clearly intended to mean. In a couple of places, I got so confused that I had to basically throw up my hands and admit defeat. I’ve drawn attention to those passages in my detailed comments below, and made various suggestions for improving the text, but I’d strongly encourage the authors to make their own careful pass(es) through the manuscript and ensure that the writing is as clear and correct as possible. By way of a couple of general suggestions, the authors sometimes write *Placerias* with a possessive apostrophe, which I don’t particularly mind but some purists might find objectionable. The manuscript tends to use “length” for all distance measurements, regardless of orientation, but in some cases it might be clearer to refer to a measurement as a width or a depth.

I have some minor comments on specific passages within the manuscript, as follows, and I’ve made some additional comments on the accompanying PDF.

(pp. 3-4, lines 124-125) Particularly given that the paper doesn’t include a phylogenetic tree, it would be good to state explicitly that placeriines and stahleckeriines are subclades within Stahleckeriidae.

(pp. 4-5, lines 174-175) The description of the edge of the caniniform process as “scraper-like” is a little inapt, as a scraper’s edge is drawn across a surface whereas the edge of the caniniform process presumably pressed into food items. Perhaps “blade-like” would be preferable. More precision about the position and extent of the edge would also be helpful. Is it just the anterior margin of the caniniform process that forms an edge of the type described?

(p. 5, line 183) The two morphs are referred to as “larger” and “smaller” here, but it’s just the size of the caniniform process (not the whole body) that differs between the two, correct? Phrases like “large-caniniform morph” and “small-caniniform morph” would capture this better. Later in the paragraph, I’m not sure what is meant by “complete sets of bones”.

(pp. 5-6, lines 223-224) Was UCMP 27370 the only maxilla mentioned (by specimen number) by Camp and Welles that was not included in the present study? Or were there others? Providing a clear answer to this question would partially address the issue of overlap between the C&W sample and the one in the present study.

(p. 6, lines 251-252) I assume the two UCMP 27553 maxillae are about the same size, but this would be worth stating explicitly.

(p. 6, line 260) It would also be worth stating what measurement of the proximal condyles is a proxy for overall fibula size, and whether it’s specifically a proxy for fibula length or for something else.

(p. 6, line 268) A little more description of the position and orientation, and perhaps also the function and general nature, of “the large cavity at the proximal end of the alveolus” would be helpful. Also, would something like “dorsal apex” (if that’s where the cavity is) be preferable to “proximal end”? Later in the paragraph, “nominal measurements” seems like the wrong phrase – something like “qualitative features” would work better.

(p. 7, line 292) The point labelled “mxs” is the anterior end of the maxillary suture, which should probably be noted.

(p. 8, lines 334-339) It would be helpful to explain a bit more about the “two predefined groups”. Predefined on what basis? Also, the caption to Figure 7 indicates that the only variables that were used as input for the PCA were the DV, ML and PAP distances, but it would be good to mention this point here as well.

(p. 9, lines 385-386) I assume the second principal component (“Dim2”, although it would be more conventional to write “PC2”), which separates the two clusters, was dominated by the DV length measurement, but this should be made clear.

(p. 10-11, lines 435-442) I don’t see how it could really be simultaneously true that (1) the tooth is in the same location relative to features such as the premaxillary and jugal sutures in both the small and large morphs, and (2) the increase in DV length in the large morph affects the entire maxilla, not just the caniniform process. If the increase in DV length is distributed across the entire height of the maxilla, then surely the tooth *must* end up farther from the premaxillary and jugal sutural areas in the large morph than in the small one? Conversely, even if the increase in DV length does affect the whole height of the maxilla, the absolute value of the “ttc” measurement must still tend to be much greater in the large morph than in the small one. Given that discrepancy, why doesn’t ttc show bimodality? Is it being scaled to DV length, perhaps? More explanation of these puzzling points seems necessary.

(p. 11, lines 455-457) If four of 32 maxillae “lack any sign of tooth eruption or socket”, does that mean that the other 28 maxillae all have sockets containing erupted teeth?

(p. 11, lines 483-486) The argument being made here seems like a bit of a stretch. The pathologies are only occasional, and the growth variations that produce ribbing and longitudinal grooving affect the root rather than the crown of the tusk, so it’s possible that crown shape was indeed under selection pressure. I think the very small size of the tusk, mentioned in the Discussion, is a much better reason to think that this structure was essentially vestigial.

(p. 12, lines 491-492) I assume the statement that the tooth dimorphism “does not correspond” to the caniniform process dimorphism means that some specimens with large caniniform processes have small tusks, and vice-versa, but it would be worth spelling this out.

(p. 12, lines 504-505) In what sense does individual variation between specimens not “correlate with two consistent morphs of premaxillae”? Is this supported by some kind of analysis, or is it just that two morphs aren’t apparent by visual inspection?

(p. 13, lines 542-543) I don’t follow the reasoning here, or even really understand what is meant by the possible bimodality of the MMC measurements being “based on proportion rather than just absolute size”. Please explain this more clearly!

(p. 13, lines 567-569) I’d say that the fibula comprising two broken sections separated by matrix and plaster shouldn’t be included among the specimens judged complete enough for the tibial condyle to distal condyle distance to be worth measuring, so there are really only eight such specimens. Later in the paragraph, the reasoning about “positively allometric” features is difficult to follow. It’s clear that diaphyseal length is being suggested to be positively allometric with respect to epiphysis size (i.e. diaphyseal length should increase faster than epiphysis size as overall body size increases), but less clear how dorsoventral midshaft diameter and other measurements fit into the argument, or how positive allometry might account for the separation between clusters seen in Figure 14c.

(p. 14, lines 630-632) What needs to be explained is not just “variance”, but more specifically bimodality, and it would be better to use that term.

(p. 15, line 660) If possible, please be more specific about what “signs of arrested growth” are present.

(p. 17, lines 769-770) The phrase “positionally similar dimorphic features… that can be classified as weaponry” dances coyly around the fact that the dimorphic features are caniniform processes in *Placerias*, and tusks in elephants, hippopotami and walruses. It would be preferable to be up front about this obvious discrepancy, which doesn’t really detract from the argument being made here.

(p. 18, lines 792-794) If the advantage of having laterally projecting tusk tips in male walruses is known, it would be good to mention it here, given that a corresponding advantage might have applied to the caniniform process of *Placerias*.

(p. 19, line 850) *Very* minor point, but my name is given incorrectly in the listing for ref. 9 – it should be “Sullivan C”, not “Sullivan G” (that would be my brother Graham!).
