# Supplementary material for: Quantitative evidence for dimorphism suggests sexual selection in the maxillary caniniform process of Placerias hesternus
Source: PLoS One. 2024 May 31;19(5):e0297894. doi: 10.1371/journal.pone.0297894 (PMC11142433; doi:10.1371/journal.pone.0297894)
Supplement: Attachment — Submitted filename: PlaceriasResponseToReviewers.docx [file pone.0297894.s007.docx]

**Review – Pinto et al., “Quantitative evidence for dimorphism suggests sexual selection in the maxillary caniniform process of *Placerias hesternus*”**

Dear C. Sullivan,

Thank you for the attentive and constructive comments on our manuscript. Below we address your suggestions one by one, and we think they contributed greatly to improve the clarity of our paper. We would also like to note that, while double checking values and results in the review process, we caught a minor mistake in our previous R code, which was repeated for the analysis of multiple characters. Specifically, we were using a command that didn't provide the correct sample size for the calculation of AICc. Correcting that mistake led to changes in model comparison, as can be observed in Table 1 of the manuscript document with tracked-changes. However, those changes were minor and did not affect the interpretation of the results.

**Review by Corwin Sullivan**

Sexual dimorphism has been repeatedly suggested in various dicynodont synapsids, and Pinto et al. have produced a worthy addition to this literature by making a strong case for dimorphism in the caniniform process of *Placerias hesternus*. The manuscript is well argued and well illustrated, and the evidence adduced for interpreting the caniniform process as a sexually dimorphic structure is compelling. On this basis, I’m delighted to recommend publication of the paper in *PLOS ONE*, following some minor revisions.

The paper ends by suggesting that the caniniform process was not just a dimorphic structure, but more specifically a weapon as opposed to an ornamental feature. This is perfectly plausible, but I think the argument could be greatly strengthened by more explicitly considering *how* one *Placerias* could have used its caniniform process to attack another. How well would the caniniform process have lent itself, for example, to stabbing and/or slashing? Another point to be made in this connection is that, if the caniniform process took over the feeding and display functions of the tooth as suggested earlier in the Discussion, it may have taken over a combat-related function as well.

Change addressed. Material is added to the end of the Discussion about the cone shaped tips of the large morph caniniforms being potentially more ideal for stabbing than slashing, with the head held high and the face pointing dorsally supported by robust neck muscles during combat, as is the case in walruses. The caniniform is also more explicitly stated to likely be taking over a previous combat related function of the tusk, which was implied by its likely role as a weapon, but not outright stated. However, we do not include specifics details of caniniform use in combat as this would be speculative at this point. That said, our work here could be used to generate hypotheses to be tested in future work, which would then add evidence for the use of the caniniforms as combat weapons. Nevertheless, we included additional inferences from behavior in animals like walruses, though it is worth noting that even in these living systems, there is little work on the details of tusk combat behavior, and there doesn’t appear to be a very specific single-use case of combat interactions (i.e. in different circumstances or depending on individual there could be differing amounts of stabbing/slashing, etc.), so we have not gone into extreme detail regarding potential behavior in *Placerias.*

The authors suggest that the morph with the large caniniform process was likely male rather than female, but are somewhat noncommittal on this point. I think a more definite case could be made that the large-caniniform morph was almost certainly the male, based not only on analogy to modern mammals but also on the distribution of sexually dimorphic armament more or less throughout the animal kingdom. Furthermore, males almost always have less investment in the offspring than females, and therefore are much more likely to have a strong incentive to maximise their mating opportunities by engaging in violent competition.

We added a sentence about the general trend of males having more intrasexual competition and investing less in offspring in mammals, the living representatives of synapsids. However, examples of the opposite trend exist in amniotes, for example, sexual size dimorphism is female biased in squamates and many clades of birds, including birds-of-prey in which females use talons and beak to defend territory. Hence, we consider it important to not over commit on this point. Furthermore, the use of caniniform as a weapon is already somewhat speculative and the respective sex of each morph doesn’t significantly change our interpretation of their dimorphism or potential behavior.

The manuscript devotes too much space, attention and analysis to cases, like that of the distance between the anterior tip of the premaxilla and the nasal process, in which the distribution of data for a measurement appears somewhat bimodal when inspected visually but statistical support for bimodality is lacking. The visual impression of bimodality is never very clear, at least to me, and in my opinion deserves to be mentioned only briefly. This aspect of the paper gives the impression that the authors are trying to wring (even) more dimorphism out of their data than is really there.

Change addressed. The sections dealing with the premaxillae, quadrates, and fibulae have been reworked to only draw attention to the fact that some of the distributions have two visually apparent peaks, but are perhaps not distinct enough, or have too low of a sample size, to be statistically recognized as dimorphic. The nature of the proportional variations in these elements has also been further clarified- while none show statistically significant evidence for dimorphism, details of their individual variation are still noted here for clarity on how they differ from the sexually selected variance of the caniniform.

It seems odd to me that the “dorsoventral length”, “mediolateral length” and “proximal anteroposterior length” measurements that lie at the core of the quantitative analysis of the maxilla’s proportions are actually not strictly dorsoventral, mediolateral and anteroposterior, but rather are all oblique measurements between specific landmarks (whereas I would expect a dorsoventral measure of an element to be the distance along the dorsoventral axis from the dorsalmost point to the ventralmost point, as if the measurement were being taken with giant calipers). An easy fix for this would be to simply rename the measurements, so that “dorsoventral length”, for instance, could become something like “jugal-caniniform length”.

Change addressed. Maxillary length labels are changed as follows:

dorsoventral length -> jugal-caniniform process dorsoventral (jcp DV) length

mediolateral length -> jugal-pterygoid pit mediolateral (jpp ML) width

proximal anteroposterior length -> jugal-premaxillary anteroposterior (jpm AP) length

(But still need to modify the figures)

The paper is nicely illustrated, and the writing is generally serviceable but sometimes vague and difficult to follow. There are a lot of sentences that contain grammatical slip-ups and/or don’t quite say what they’re clearly intended to mean. In a couple of places, I got so confused that I had to basically throw up my hands and admit defeat. I’ve drawn attention to those passages in my detailed comments below, and made various suggestions for improving the text, but I’d strongly encourage the authors to make their own careful pass(es) through the manuscript and ensure that the writing is as clear and correct as possible.

By way of a couple of general suggestions, the authors sometimes write *Placerias* with a possessive apostrophe, which I don’t particularly mind but some purists might find objectionable.

Change addressed. Instances of the possessive “Placerias’” have been removed.

The manuscript tends to use “length” for all distance measurements, regardless of orientation, but in some cases it might be clearer to refer to a measurement as a width or a depth.

Change addressed.

mediolateral length -> jugal-pterygoid pit mediolateral (jpp ML) width.

I have some minor comments on specific passages within the manuscript, as follows, and I’ve made some additional comments on the accompanying PDF.

All changes suggested within the PDF have been addressed.

(pp. 3-4, lines 124-125) Particularly given that the paper doesn’t include a phylogenetic tree, it would be good to state explicitly that placeriines and stahleckeriines are subclades within Stahleckeriidae.

Change addressed, placeriines and stahleckeriines have been clarified as stahleckeriids.

(pp. 4-5, lines 174-175) The description of the edge of the caniniform process as “scraper-like” is a little inapt, as a scraper’s edge is drawn across a surface whereas the edge of the caniniform process presumably pressed into food items. Perhaps “blade-like” would be preferable. More precision about the position and extent of the edge would also be helpful. Is it just the anterior margin of the caniniform process that forms an edge of the type described?

Change addressed, “blade-like” has been used, and a sentence is added about the posterior surface not having the edge has been added.

(p. 5, line 183) The two morphs are referred to as “larger” and “smaller” here, but it’s just the size of the caniniform process (not the whole body) that differs between the two, correct? Phrases like “large-caniniform morph” and “small-caniniform morph” would capture this better. Later in the paragraph, I’m not sure what is meant by “complete sets of bones”.

Change addressed, “large-caniniform morph” and “small-caniniform morph" have been added (and “large morph” and “small morph” have been clarified to be effectively equivalent to these terms for our purposes and used thereafter.). “Complete sets of bones” has been replaced with “well-represented bones”, to convey that while other bones could be measured, the three we chose are among the best represented in the assemblage.

(pp. 5-6, lines 223-224) Was UCMP 27370 the only maxilla mentioned (by specimen number) by Camp and Welles that was not included in the present study? Or were there others? Providing a clear answer to this question would partially address the issue of overlap between the C&W sample and the one in the present study.

Change addressed, UCMP 27370 is the only maxilla mentioned by specimen number by C&W not included in this study, assuming that 27319 and 28389 are indeed mislabeled. This has been clarified in the text.

(p. 6, lines 251-252) I assume the two UCMP 27553 maxillae are about the same size, but this would be worth stating explicitly.

Change addressed.

(p. 6, line 260) It would also be worth stating what measurement of the proximal condyles is a proxy for overall fibula size, and whether it’s specifically a proxy for fibula length or for something else.

Change addressed. Dorsoventral length of the proximal condyle is used as a proxy for fibula length.

(p. 6, line 268) A little more description of the position and orientation, and perhaps also the function and general nature, of “the large cavity at the proximal end of the alveolus” would be helpful. Also, would something like “dorsal apex” (if that’s where the cavity is) be preferable to “proximal end”? Later in the paragraph, “nominal measurements” seems like the wrong phrase – something like “qualitative features” would work better.

Change partially addressed. Information has been added on the position and orientation of the proximal end of the alveolus (name is changed to “cavity proximal to the alveolus” for clarification, as it is not technically part of the alveolus, but is connected to it in some individuals), and its likely function as the dental chamber. It has not been renamed the “dorsal apex” as suggested, as it is not the dorsal-most point on the maxilla.

The use of “nominal measurements” has been retained, as it is appropriate following definitions of measurement theory (see Voje *et al*. 2023, Measurement theory and Paleobiology, TREE, <https://doi.org/10.1016/j.tree.2023.08.005>).

(p. 7, line 292) The point labelled “mxs” is the anterior end of the maxillary suture, which should probably be noted.

Change addressed.*

(p. 8, lines 334-339) It would be helpful to explain a bit more about the “two predefined groups”. Predefined on what basis? Also, the caption to Figure 7 indicates that the only variables that were used as input for the PCA were the DV, ML and PAP distances, but it would be good to mention this point here as well.

Change partially addressed. The number of groups is preselected by the program input, and so not defined by any specific attribute. The assessment of if there are actually two groups comes from the inspection of the clusters generated with this ruleset, to see if they actually lack overlap along some measured dimension, or do overlap and are only made into clusters because the program is being made to create two clusters. This has been clarified in the Methods. The DV, ML, and PAP distances are used for the maxillae, which is clarified in the results for the maxillae (just before the figure caption), but different measurements are used for the PCAs of other elements, so it should not be stated here in the methods that the DV, ML, PAP values are the only ones used, as this only applies to the maxillae PCA.

(p. 9, lines 385-386) I assume the second principal component (“Dim2”, although it would be more conventional to write “PC2”), which separates the two clusters, was dominated by the DV length measurement, but this should be made clear.

Change addressed.

(p. 10-11, lines 435-442) I don’t see how it could really be simultaneously true that (1) the tooth is in the same location relative to features such as the premaxillary and jugal sutures in both the small and large morphs, and (2) the increase in DV length in the large morph affects the entire maxilla, not just the caniniform process. If the increase in DV length is distributed across the entire height of the maxilla, then surely the tooth *must* end up farther from the premaxillary and jugal sutural areas in the large morph than in the small one? Conversely, even if the increase in DV length does affect the whole height of the maxilla, the absolute value of the “ttc” measurement must still tend to be much greater in the large morph than in the small one. Given that discrepancy, why doesn’t ttc show bimodality? Is it being scaled to DV length, perhaps? More explanation of these puzzling points seems necessary.

Change addressed. The paragraph discussing this has been reworked, to clarify that the small absolute value of the ttc lengths leads to higher impact on the distribution of lengths by the gaps within each morph, which are (in terms of percentage) larger than the gap between morphs.

(p. 11, lines 455-457) If four of 32 maxillae “lack any sign of tooth eruption or socket”, does that mean that the other 28 maxillae all have sockets containing erupted teeth?

Change addressed. 20 of the maxillae have sockets containing erupted teeth, but the other 8, despite missing teeth, also have visible sockets, indicating the tooth erupted at some point, and then likely fell out post-mortem.

(p. 11, lines 483-486) The argument being made here seems like a bit of a stretch. The pathologies are only occasional, and the growth variations that produce ribbing and longitudinal grooving affect the root rather than the crown of the tusk, so it’s possible that crown shape was indeed under selection pressure. I think the very small size of the tusk, mentioned in the Discussion, is a much better reason to think that this structure was essentially vestigial.

Change addressed. A point about the small size of the tusk has been added to this section, as well as a point about the variance in crown shape providing additional evidence for lack of selection pressure.

(p. 12, lines 491-492) I assume the statement that the tooth dimorphism “does not correspond” to the caniniform process dimorphism means that some specimens with large caniniform processes have small tusks, and vice-versa, but it would be worth spelling this out.

Change addressed.

(p. 12, lines 504-505) In what sense does individual variation between specimens not “correlate with two consistent morphs of premaxillae”? Is this supported by some kind of analysis, or is it just that two morphs aren’t apparent by visual inspection?

Change addressed. Two morphs are not apparent by visual inspection. Clustering (without pg lengths) shows two potential morphs of premaxilla, but these are not distinct enough to be supported by any stats. The statement about “correlating with consistent morphs” has been replaced with these points.

(p. 13, lines 542-543) I don’t follow the reasoning here, or even really understand what is meant by the possible bimodality of the MMC measurements being “based on proportion rather than just absolute size”. Please explain this more clearly!

Change addressed. This paragraph has been reworded and split, to say that there are two potential modules on the quadrate, the MMC and the LMC. While the first had some suggestions of biomodality the latter does not, which raises the possibility that the population does not consist of two different quadrate size distributions, but instead two MMC size distributions, with more typical unimodal individual variation in the LMC.

(p. 13, lines 567-569) I’d say that the fibula comprising two broken sections separated by matrix and plaster shouldn’t be included among the specimens judged complete enough for the tibial condyle to distal condyle distance to be worth measuring, so there are really only eight such specimens. Later in the paragraph, the reasoning about “positively allometric” features is difficult to follow. It’s clear that diaphyseal length is being suggested to be positively allometric with respect to epiphysis size (i.e. diaphyseal length should increase faster than epiphysis size as overall body size increases), but less clear how dorsoventral midshaft diameter and other measurements fit into the argument, or how positive allometry might account for the separation between clusters seen in Figure 14c.

Change addressed. The passage about the broken and repaired fibula is removed, and it is discarded from the tc to dc length distribution and histogram. Traits in which the 2 larger fibulae separate out from the rest of the sample are related to diaphysis size (length and diameter), so the implication is that the largest fibulae have proportionately larger diaphyses. The distribution of tc to dc and the clusters seen in 14a and 14c are both driven by tc to dc, and here we interpret the distribution as a normal one with two outliers instead of a bimodal one, as is reflected in the stats.

(p. 14, lines 630-632) What needs to be explained is not just “variance”, but more specifically bimodality, and it would be better to use that term.

Change addressed.

(p. 15, line 660) If possible, please be more specific about what “signs of arrested growth” are present.

Change addressed. Evidence of maturity among all sampled *Placerias* individuals is mainly attributed to the universal presence of extensive secondary remodeling.

(p. 17, lines 769-770) The phrase “positionally similar dimorphic features… that can be classified as weaponry” dances coyly around the fact that the dimorphic features are caniniform processes in *Placerias*, and tusks in elephants, hippopotami and walruses. It would be preferable to be up front about this obvious discrepancy, which doesn’t really detract from the argument being made here.

Change addressed.

(p. 18, lines 792-794) If the advantage of having laterally projecting tusk tips in male walruses is known, it would be good to mention it here, given that a corresponding advantage might have applied to the caniniform process of *Placerias*.

Change addressed.

(p. 19, line 850) *Very* minor point, but my name is given incorrectly in the listing for ref. 9 – it should be “Sullivan C”, not “Sullivan G” (that would be my brother Graham!).

Change addressed.
